# School Refusal Behaviors: The Roles of Adolescent and Parental Factors

**DOI:** 10.1177/01454455241276414

**Published:** 2024-08-26

**Authors:** Junwen Chen, Celina Feleppa, Tingyue Sun, Satoko Sasagawa, Michael Smithson, Liana Leach

**Affiliations:** 1 Australian National University, Canberra, ACT, Australia; 2 Flinders University, Adelaide, SA, Australia; 3 Mejiro University, Shinjuku, Tokyo, Japan

**Keywords:** school refusal behaviors, age, emotion dysregulation, anxiety and depression, parental rearing styles, parental and adolescent factors

## Abstract

School refusal behaviors in adolescents have deleterious immediate and long-term consequences and are associated with mental ill-health such as anxiety and depression. Understanding factors that place youth at higher risk of school refusal behavior may assist in developing effective management approaches. We investigated parental and adolescent factors that may be associated with school refusal behaviors by specifically focusing on the role of parental and adolescent emotion dysregulation, their anxiety and depression, and parental rearing style. First, we hypothesized that adolescents with school refusal behaviors, as well as their parents, will report higher levels of emotion dysregulation, anxiety, and depression compared to their counterparts without school refusal behaviors. Furthermore, we hypothesized that multivariate models testing the role of parental and child factors concurrently will show that parental (emotion dysregulation, anxiety and depression, and rearing styles) and adolescent (emotion dysregulation, anxiety, and depression) factors are associated with school refusal behaviors. One hundred and six adolescents aged 12 to 18 years and their parents completed an online questionnaire measuring both parental and adolescent emotion dysregulation, anxiety, depression, parental rearing styles, and adolescents’ school refusal behaviors. Adolescents with school refusal behaviors reported greater anxiety and depression, with their parents showing greater emotion dysregulation. Multivariate analyses showed that parental emotion dysregulation and adolescent age were associated with school refusal behaviors independently. Future management for school refusal behaviors should consider age-tailored approaches by incorporating training for parental emotion regulation skills.

## School Refusal Behaviors

School refusal behaviors refer to behaviors aimed at avoiding school attendance or any difficulties attending school or remaining in classes for an entire day (e.g., late to school, leaving school early), often due to anxiety-related or other emotional (e.g., depression) difficulties (Elliott & Place, 2019; Ingul et al., 2019). School refusal behaviors often manifest at certain transition points (e.g., from primary school to secondary school), where the youth^1^ face greater academic demands and more complex social relationships (Garfi, 2018).

School refusal behaviors have detrimental effects on both school and post-school functioning. Poor school attendance is a strong indicator of school disengagement, lower academic achievement, higher dropout rates, and problematic social-emotional development (Allen et al., 2018; Kearney, 2022). It is also highly correlated with mental health problems such as anxiety, depression, suicide attempts, violence, and substance use and abuse (Allen et al., 2018; Ingul et al., 2019). In the longer term, regular school absences are linked to negative outcomes in adulthood, including lower income, marital problems, unemployment, and diminished mental health (Allen et al., 2018; Kearney, 2022). Given the adverse impact of school refusal, a thorough understanding of factors that place youth at a higher risk of school refusal behavior is needed to develop effective management approaches.

## Parental and Youth Anxiety, Depression, and Emotion Dysregulation

The interdisciplinary model (Kearney, 2008) proposes that both parental and youth factors are key contributors to school refusal behaviors in children and adolescents, among which, parental and youth psychopathology (e.g., anxiety and depression) play an important role. Youth with higher emotional instability (i.e., higher tendency to experience anxiety, sadness, etc.) are more likely to interpret situations as being threatening and therefore are more vulnerable to school refusal behaviors. Research has shown that anxious school refusers reported a higher rate of anxiety (5%–18%) and depression (14%) compared to their non-anxious counterparts (Ingul et al., 2019). Likewise, 53% to 78% of parents with school-refusing youths experience anxiety and depressive disorders (Martin et al., 1999).

In addition to parental and youth anxiety and depression, problematic emotion regulation, also understood as emotion dysregulation, has been suggested as a key risk factor in a more recent framework for school refusal (Ingul et al., 2019). Emotion regulation refers to the way individuals influence their experience of emotions including what, when, and how emotions are expressed (Mennin et al., 2002). Emotion regulation includes two key strategies, namely cognitive reappraisal and expressive suppression (Gross & John, 2003). Cognitive reappraisal involves changing the way one thinks about their emotions, whilst expressive suppression involves inhibiting emotions by hiding them from others. Cognitive reappraisal is associated with increased psychological functioning, hence it is considered a “healthy” emotion regulation strategy. In contrast, expressive suppression is viewed as a problematic emotion regulation strategy as it is associated with increased negative affect in the long term. A study by E. K. Hughes et al. (2010) found that school refusing youths (10–14 years old) engaged in more expressive suppression and less cognitive reappraisal compared to regular attenders. However, the high comorbid rates of anxiety disorders (33%–43%) in the school refusing youths made it difficult to determine whether the differences were due to anxiety disorders or school refusal behaviors. This highlights the importance of investigating the extent to which youths’ emotional regulation and psychopathology each independently are associated with school refusal.

Research suggests that youths learn behaviors by observing how their parents respond to emotional stimuli (Bariola et al., 2012; Morris et al., 2017). For example, Bariola et al (2012) found that mothers’ expressive suppression predicted their child’s (9–19 years old) expressive suppression. Although no direct evidence on school refusal behaviors has been provided to date, parental emotion regulation (e.g., emotion expression) has been suggested to influence children’s prosocial behaviors (Morris et al., 2017). Thus, parents’ emotional regulation is an important under-researched factor which we expect also plays a role in youths’ school refusal behaviors.

## Parental Rearing Styles

Problematic parental rearing style is another factor that may contribute to school refusal, although like parental emotional regulation, there is a paucity of evidence testing this association (Kearney, 2008). Parental rearing style has been defined as attitudes from parents towards their child that create an emotional climate within which the parent’s behaviors are expressed (Darling & Steinberg, 1993). Parental over-control, also termed parental restrictiveness, involves excessively regulating, intruding, and discouraging youth’s activities and problem-solving behaviors. In contrast, nurturance is characterized by parents’ warmth, acceptance, and responsiveness toward their child. Parental acceptance-rejection theory has argued that there is a need for positive responses from parental figures for children and adolescents to feel comfort, support, and nurturance (Khaleque & Rohner, 2012). When this need is unmet, through problematic rearing styles such as over-control or rejection, youths tend to develop specific emotional temperaments (e.g., anxiety and sadness), or problematic behaviors (e.g., school refusal behaviors; Yaqoob & Tahira, 2020).

To our knowledge, only one study has directly examined the relationship between parental rearing style and school refusal function in children aged 5 to 13 years (Yaqoob & Tahira, 2020). The results showed that rejective parenting style (e.g., ignoring the child’s demands) was associated with children’s avoidance of anxiety-provoking situations at school. Furthermore, both controlling and rejective parental styles predicted children’s anxiety. However, this study examined the relationships between parental rearing styles and children’s anxiety, as well as parental rearing styles and school refusal separately. The current study aimed to extend these findings by examining whether parental rearing styles, including both over-control and nurturance (i.e., lower levels of rejection), explain youth’s school refusal behaviors independently of parental and youth psychopathology.

## The Interplay Between Parental and Youth Factors

Frequent school absences are often driven by more than one factor (Allen et al., 2018). However, only a limited number of studies have examined the interplay between the parental and adolescent factors discussed above in school refusal behaviors. P. M. Hughes et al. (2020) examined how parental (i.e., anxiety and depression, parental psychological control) and adolescent factors (i.e., emotion regulation, internalizing, and externalizing problems) predicted school refusal behaviors in 184 adolescents (*M*_age_ = 15.26 years) who sought help for psychological problems. The results showed that parental psychological control was significantly associated with adolescent expressive suppression (but not cognitive reappraisal) and externalizing problems such as aggression (but not internalizing problems such as anxiety and depression). There were no other significant relationships. However, this study only included adolescents who participated in a residential treatment program, which limited the generalization of its findings. In addition, the study only focused on parental anxiety and depression without addressing parental emotion regulation. There is scope for further research to examine both the independent contribution and the intersection between parent and adolescent factors (both in terms of psychological mechanisms and parenting practices) on school refusal.

## The Present Study

The present study aimed to examine the role of adolescent and parental factors in school refusal behaviors by building on the theoretical frameworks of school refusal behaviors (Ingul et al., 2019; Kearney, 2008). First, we tested whether adolescents with school refusal behaviors and their parents report higher levels of emotion dysregulation, anxiety, and depression compared to their counterparts without school refusal behaviors. Differences in parental rearing style (nurturance and restrictiveness) were also tested between parents of adolescents with and without school refusal behaviors. Second, we used multivariate models to examine whether parental (emotion dysregulation, anxiety and depression, and rearing styles) and adolescent (emotion dysregulation, anxiety, and depression) factors were independently or jointly associated with school refusal behaviors. Third, we tested potential interactive effects between parents and child factors. The identification of both parental and adolescent factors that contribute to school refusal behaviors is critical to informing novel and effective approaches that support families in managing school refusal behaviors in adolescents.

## Methods

### Participants

Adolescents aged 12 to 18 years old were recruited from South Australian public (*n* = 6) and private (*n* = 4) high schools via an in-school advertisement (see details in Procedure), together with their parents. There were 207 parents and 178 adolescents who initially participated in the study. Only 106 matched data pairs of the adolescent (*M*_age_ = 14.79 years old, *SD* = 1.78, males = 44) and their parent (*M*_age_ = 47.49 years old, *SD* = 5.61, males = 29) were included in the analysis.

All but two public schools had above-average community socio-educational advantages relative to national data (ACARA, 2021). Thirty participants (30 adolescents and their parents) were allocated to the school refusal behaviors group, and 76 to the non-school refusal behaviors group, based on the criteria deemed by the DECD (2017–2020) (Supplemental Material 1, also see the School Attendance section).

### Materials

Participants completed questionnaires assessing their anxiety and depression, emotion regulation strategies (both parents and their child), as well as parental rearing styles, and adolescents’ school refusal behaviors.

#### Depression Anxiety Stress Scales-21 (DASS-21)

The anxiety and depression subscales of the DASS-21 (Lovibond & Lovibond, 1995) measured symptoms of anxiety and depression in the past week in both parents and their child. Items are rated from 0 (“did not apply to me at all”) to 3 (“applied to me very much, or most of the time”). The two subscales have high internal consistency (α_DASS-21-Depression_ = .96 and α_DASS-21-Anxiety_ = .89) and the DASS-21 as a whole has good validity. The subscales for this study also had high internal consistency for parents (α_DASS-21-D_ = .91 and α_DASS-21-A_ = .85) and youths (α_DASS-21-D_ = .88 and α_DASS-21-A_ = .86). A composite score of depression and anxiety was created, using the means of the standardized scores of depression and anxiety, given the moderately high correlations between them in both adolescents and parents (*r* = .66–.68, *p*’s < .01).

#### Emotion Regulation Questionnaire (ERQ)

The ERQ (Gross & John, 2003) measures parental emotion regulation strategies of cognitive reappraisal and expressive suppression. The 10-item ERQ was rated from 1 (“strongly disagree”) to 7 (“strongly agree”). For this study, items on the cognitive reappraisal subscale were reversed to obtain a score of emotion dysregulation. The ERQ has acceptable internal consistencies (α = .73–.79) and test-retest reliability (*r* = .69). It also has good convergent and discriminant validity. The internal consistency in the current study was good (cognitive reappraisal: α = .83; expressive suppression: α = .73).

#### Emotion Regulation Questionnaire for Children and Adolescents (ERQ-CA)

The ERQ-CA (Gullone & Taffe, 2012) is a revised version of the ERQ to measure emotion regulation strategies in children and adolescents. The 10-item inventory measures expressive suppression and cognitive reappraisal using a scale from 1 (“strongly disagree”) to 5 (“strongly agree”). Items measuring cognitive reappraisal were reversed to obtain a score of emotion dysregulation. It has good convergent and construct validity (Gullone & Taffe, 2012). The internal consistency in the current study was high for cognitive reappraisal subscale (α = .88) and good for expressive suppression subscale (α = .71).

#### Child Rearing Practices Report (CRPR) (Modified Version)

The CRPR is a modified version of the original CRPR, which measured parental child rearing attitudes and values (Rickel & Biasatti, 1982). The 40-item CRPR (Modified) assesses restrictiveness (i.e., over-control, “I control my child by warning them about the bad things that can happen to them”) and nurturance (i.e., lack of rejection, “I express my affection by hugging, kissing, and holding my child”), using a 6-point scale ranging from 1 (“not at all descriptive of me”) to 6 (“highly descriptive of me”). The two subscales have good internal consistencies (α = .84–.85). The internal consistency in the current study was high for both restrictiveness (α = .84) and nurturance (α = .80) subscales.

#### School Attendance

In line with previous studies (see Supplemental Material 2) and the guidelines for school non-attendance by the DECD (2017–2020), a student who is absent for 10 days or more in a term (average of 1 day per week) for any reason beyond physical illness is identified as having school refusal behavior (see Supplemental Material 1).

Attendance data for adolescent participants were provided by the participating high schools, including full and half days absent. We counted the number of school days on which a school refusal behavior occurred (i.e., missing school or leaving school early/arriving late). Thus, a half-day absence (i.e., leaving school early or arriving late) was considered a day that the student engaged in school refusal behaviors. Schools also indicated whether the absences were explained or unexplained, and whether explained days were due to illness or another reason. Students completed a school attendance questionnaire on how many days (1) they have missed school in a 4-week period, (2) they have been late or left school early, and (3) were missed for illness or feelings of anxiety or worry. Days missed due to illness were not considered unless there was a discrepancy between the attendance data gathered from the school (i.e., it was recorded the student was ill) and the responses provided by the student (i.e., they reported feeling too anxious to attend school). In such cases, the student’s response was included in determining school refusal behavior based on their specified reasons (e.g., feelings of anxiety). Any days absent that were unexplained or were due to mental health challenges were also included as days absent. This combination of both school administrative attendance data and student-reported data provides a more accurate assessment of school refusal than either method of data collection alone.

### Procedure

All high schools were first contacted through email and phone calls to introduce the study. When a school expressed its interest, a researcher arranged a meeting with the school to explain the study and answer any questions. After receiving approval from the school for participation, the study was advertised via school-wide email or school newsletter. The researcher delivered the information package to schools for teachers to pass it to interested students in their classes. Participation was voluntary. All participants (adolescents and parents) gave consent before participating in the study. Following participation, participants could be entered in a draw to win one A$30 gift voucher if they wished.

Upon receiving signed consent forms, all students completed the measures online at school during their class time with a teacher and the researcher present. The researcher provided verbal instructions to the students about the survey. Parent participants were emailed a link to complete the questionnaires at home in their own time. Parent and adolescent data were matched by a shared code allocated to them. This study was approved by the Flinders University Social and Behavioral Research Ethics Committee, the Department for Education and Child Development South Australia, and Catholic Education South Australia.

## Results

### Preliminary Analysis

All parent and adolescent variables presented skew and kurtosis within normal distribution. Age and gender differences (i.e., key demographics) in the adolescents with and without school refusal behaviors, and their parents were tested. Results are shown in Table 1. There was a significant age difference between the adolescents with and without school refusal behaviors (with school refusers being older). As such, age was controlled for in the subsequent regression analyses.

**Table 1.** Differences Between Adolescents With and Without School Refusal Behaviors and Parents.

Variables	SRB (*M*, *SD*) (*N* = 30)	Non-SRB (*M*, *SD*) (*N* = 76)	χ^2^(*df*) or *t*(*df*)	*p*	Effect size
**Adolescents**					
Gender			χ^2^(1) = 1.15	.38	–
Female (*N*, %)	20 (66.7%)	42 (55.3%)			
Male (*N*, %)	10 (33.3%)	34 (44.7%)			
Age	15.67 (1.75)	14.45 (1.78)	*t* (104) = 3.20	.002	
Anxiety + depression	7.65 (4.33)	4.67(4.41)	*t* (104) = −3.15	.002	.68
Emotion dysregulation	31.50 (5.30)	29.57 (5.62)	*t* (104) = −1.62	.108	.35
**Parents**					
Gender			χ^2^(1) = .34	.64	-
Female (*N*, %)	23 (76.7%)	54 (71.1%)			
Male (*N*, %)	7 (23.3%)	22 (28.9%)			
Age	46.69 (7.67)	46.68 (7.60)	*t* (104) = 0.05	1.00	
Anxiety + depression	8.57 (3.37)	8.45 (3.10)	*t* (104) =−.16	.870	.04
Emotion dysregulation	30.20 (8.14)	25.41 (6.99)	*t* (104) = −3.03	.003	.65
Restrictiveness RS	48.60 (14.46)	52.11 (12.03)	*t* (104) = 1.28	.203	.28
Nurturance RS	92.57 (14.98)	96.70 (7.95)	*t* (35.6) = 1.43	.161	.40

*Note.* RS = rearing style; SRB = school refusal behaviors (i.e., 10 days or more absent); *M* = means; *SD* = standard deviation; Effect size: Cohen’s *d*.

### Differences Between Adolescents With and Without School Refusal Behaviors and Their Parents

A series of independent samples *t*-tests were conducted to test the differences between adolescents with and without school refusal behaviors and their parents on anxiety, depression, and emotion dysregulation, as well as parental rearing styles (restrictiveness, nurturance). The six *t*-tests were performed using Bonferroni adjusted alpha levels of .008 per test (.05/6) to reduce the Type I error. Adolescents with school refusal behaviors reported significantly higher levels of anxiety and depression compared to their counterparts without school refusal behaviors, with a medium effect size (see Table 1). In addition, parents of school refusing adolescents reported significantly higher levels of emotion dysregulation scores than their counterparts, with a medium effect size. No other significant differences between the two groups were found.

### Associations Between Parental and Adolescent Factors and School Refusal Behaviors

Sequential binary logistic regression analysis was employed to test the associations between parental factors (i.e., emotion dysregulation, parental anxiety and depression, and parental rearing style) and adolescent factors (i.e., emotion dysregulation, anxiety, and depression) and adolescents’ school refusal behaviors, while controlling for the adolescents’ age. Parental rearing style was tested for each subscale, namely nurturance and restrictiveness. Anxiety and depression were examined using the composite score (see Table 2).

**Table 2.** The Relationships Between Parental and Adolescent Factors and School Refusal Behaviors.

Model	Variables	*B*	*SE*	Wald’s *c*^2^	*p*	Exp (*B*)	CI	
							Lower	Upper
Step 1	Adolescent age	0.40	.13	8.72	.003**	1.49	1.14	1.94
Step 2	Adolescent age	0.44	.15	9.12	.003**	1.56	1.17	2.08
	Parental ED	0.75	.26	8.29	.004**	2.11	1.27	3.50
Step 3	Adolescent age	0.44	.15	8.82	.003**	1.55	1.16	2.08
	Parental ED	0.82	.27	9.08	.003**	2.28	1.33	3.89
	Parental depression + anxiety	−0.22	.26	0.75	.39	0.80	0.49	1.32
Step 4	Adolescent age	0.39	.15	6.57	.01*	1.47	1.10	1.98
	Parental ED	0.80	.29	7.70	.006**	2.23	1.27	3.94
	Parental depression + anxiety	−0.26	.27	0.94	.33	0.77	0.46	1.30
	Parental nurturance	−0.23	.25	0.82	.37	0.80	0.49	1.30
	Parental restrictiveness	−.039	.28	2.03	.15	0.67	0.39	1.16
Step 5	Adolescent age	0.41	.16	7.15	.007**	1.51	1.12	2.05
	Parental ED	0.79	.30	7.04	.008**	2.20	1.23	3.95
	Parental depression + anxiety	−0.30	.27	1.27	.26	0.74	0.44	1.25
	Parental nurturance	−0.21	.26	0.67	.41	0.81	0.49	1.34
	Parental restrictiveness	−0.40	.28	2.11	.15	0.67	0.39	1.15
	Adolescent ED	0.43	.26	2.61	.11	1.53	0.91	2.57
Step 6	Adolescent age	0.38	.16	5.86	.02*	1.46	1.08	1.99
	Parental ED	0.76	.29	6.69	.01*	2.14	1.20	3.81
	Parental depression + anxiety	−0.30	.27	1.24	.27	0.74	0.43	1.26
	Parental nurturance	−0.25	.26	0.97	.32	0.78	0.47	1.29
	Parental restrictiveness	−0.31	.28	1.20	.27	0.73	0.42	1.28
	Adolescent ED	0.28	.29	1.00	.32	1.33	0.76	2.32
	Adolescent depression + anxiety	0.51	.26	3.78	.05	1.66	1.00	2.77

*Note. B* = unstandardized regression coefficients; *SE* = standard error; CI = confidence interval; ED = emotion dysregulation.

* *p* < .05. **p < .01. (two-tailed tests).

Adolescent age in Step 1 and parental emotion dysregulation (in addition to Adolescent age) in Step 2 were significantly associated with adolescents’ school refusal behaviors. Adding parental anxiety and depression in Step 3, parental nurturance and restrictiveness in Step 4, and adolescent emotion dysregulation in Step 5 did not significantly improve the model fits (the likelihood-ratio tests comparing the models in Steps 2 and 3: χ^2^(1) = 0.77, *p* = .38; Steps 3 and 4: χ^2^(2) = 2.41, *p* = .30; and Steps 4 and 5: χ^2^(1) = 2.74, *p* = .10). When adolescent anxiety and depression was entered in Step 6, the model fit improved significantly (χ^2^(1) = 3.87, *p* < .05), but adolescent anxiety and depression were not significantly associated with school refusal behaviors.

These model comparisons showed that only adolescent age and parental emotion dysregulation were significantly associated with adolescents’ school refusal behaviors. To further examine whether there were joint effects of parental emotion dysregulation, and adolescent age on school refusal behaviors, the correlation between parental emotion dysregulation scores and adolescent age was examined. The result was not significant (*r* = .02, *p* = .88). Further, the interaction term between parental emotion dysregulation and adolescent age did not significantly improve the model fit (χ^2^(1) = 0.40, *p* = .53).

## Discussion

The present study investigated the role of adolescent and parental psychopathological factors (i.e., anxiety, depression, and emotion dysregulation), and parental rearing styles in school refusal behaviors across 10 public and private high schools. The study addresses an important gap in the existing literature, as to date, very little research has explored the potential for parents’ emotional regulation and child-rearing style as a critical factor in their children’s school refusal. Uniquely we explore these parental factors both in combination with and independent of children’s own mental health and emotional regulation factors.

### Differences Between Adolescents With and Without School Refusal Behaviors

Our results showed that adolescents displaying school refusal behaviors have significantly higher levels of anxiety and depression than those without school refusal behaviors. These results support the model of Kearney (2008), demonstrating the importance of youth psychopathology in school refusal behaviors. In line with Allen et al. (2018), school absences can lead to academic setbacks and disconnection from peers and supportive school staff. This disengagement diminishes the rewards of school attendance and increases anxiety, potentially resulting in chronic absenteeism and eventual dropout. In addition, our results revealed that parents of adolescents with school refusal behaviors reported significantly higher levels of emotion dysregulation than parents of adolescents without school refusal behaviors. In Ingul et al.’s (2019) framework, youth’s problematic emotion regulation (i.e., emotion dysregulation) is proposed as a risk factor for school refusal behaviors. However, the current study did not identify adolescent emotion dysregulation as a significant factor, as suggested by Ingul et al. (2019). Instead, it found that parental emotion dysregulation played a significant role in influencing adolescents’ school refusal behaviors. Our study is the first to provide empirical evidence on the relationship between parental emotion dysregulation and adolescents’ school refusal behaviors. Our results suggest that parental emotion dysregulation may intensify the challenges of managing their child’s school refusal behaviors by diminishing their ability to effectively support a distressed child.

Inconsistent with previous research (e.g., Bahali et al., 2011; Yaqoob & Tahira, 2020), parents of adolescents with school refusal behaviors did not report higher levels of anxiety, depression, and problematic rearing styles. Bahali et al. (2011) used the Beck Depression Inventory and the State-Trait Anxiety Inventory to assess depression and anxiety, rather than the Depression Anxiety Stress Scales used in the current study. Thus, a direct comparison of the results is difficult to make confidently. Furthermore, both Bahali et al. (2011) and Yaqoob and Tahira (2020) included younger children (5–13 years old) instead of adolescent samples. The differences between the assessment tools and the study samples likely led to inconsistencies.

In terms of understanding the findings for parent rearing styles, it is important to note that parents in the current study reported higher levels of nurturance (*M* = 94.64 vs. 81.6) and lower levels of restrictiveness (*M* = 50.36 vs. 72) compared to a sample of parents of children aged 4 to 12 years in the Netherlands (Krikken et al., 2012). These scores may reflect a social desirability bias given the wording of the questions. For example, questions such as “I express my affection by hugging, kissing, and holding my child” and “I let my child know how ashamed and disappointed I am when they misbehave” are typically considered to be appropriate and inappropriate behaviors, respectively, that parents should and should not be displaying toward their children in Australian culture. It has been argued that parents are always wanting to be reviewed as “good” parents, in particular mothers/female caregivers, who tend to report more positive parenting behaviors (Korelitz & Garber, 2016). As the majority of parent participants in the current study were female, this may have increased responding bias. Furthermore, a meta-analysis has found that parents’ reports on parenting behaviors were overall more favorable compared to their children’s reports. Specifically, parents saw themselves as showing more warmth and acceptance and less psychological control than their children’s perceptions (Korelitz & Garber, 2016). Thus, future studies should aim to measure parenting styles from both parents’ and youths’ points of view to determine whether the reported practices are consistent with those actually employed.

The results also revealed that adolescents with school refusal behaviors were older than their counterparts without school refusal behaviors. In addition, adolescent age was significantly and independently associated with school refusal behaviors (after controlling for other important adolescent and parent factors). Adolescence is a period of life transitions with heightened vulnerability to developing school refusal or precipitating the onset of school refusal (Ingul et al., 2019). Older adolescents may encounter increased difficulty when they engage with a more complex and demanding curriculum, experiencing more severe symptoms at a stage when high-stakes testing is becoming more pressing (Heyne et al., 2014). Future investigations should seek to identify factors that contribute to younger and older adolescents’ school refusal behaviors to assist in age-tailored management.

### The Relationships Between Parental and Adolescent Factors and School Refusal Behaviors

Our study showed that parental emotion dysregulation and adolescents’ age were significantly and independently associated with adolescents’ school refusal behaviors when all hypothesized factors were tested concurrently, but that the other parent and child mental health factors included were not. These results are inconsistent with P. M. Hughes et al. (2020), who found that adolescent expressive suppression and internalizing problems (e.g., anxiety and depression) mediated the relationship between parental psychological control (i.e., parental rearing style) and school refusal behaviors. The inconsistencies could be due to the innovative inclusion of parental emotion regulation in the investigation. The robust effects of parental emotion dysregulation may have cancelled the effects of parental rearing style as shown by P. M. Hughes et al. (2020). Parental emotional expression has been suggested to impact the development of youth’s social competence (Bariola et al., 2012; Kiel & Kalomiris, 2015). Youth may be more vulnerable to difficulties associated with social competence and adjustment, such as emotion-eliciting school attendance situations, if they learn emotion dysregulation strategies from their parents.

Consistent with P. M. Hughes et al. (2020), parental anxiety and depression were not significantly associated with adolescents’ school refusal behaviors when examining them with other parental and adolescent factors. As anxiety and depression are the products of emotion dysregulation (Gross & John, 2003), these results may indicate that parental emotion dysregulation plays a more robust role in youth school refusal behaviors, which renders the effects of parental anxiety and depression insignificant in the current sample. Research has shown that parents often have the intention to manage difficult situations for their children (Morris et al., 2017). For example, they may help their child avoid anxiety-related distress when they perceive that their child fears certain situations and believe that their child cannot cope with the situations (Hannesdottir & Ollendick, 2017). Together with the results that adolescent emotion dysregulation was not significantly associated with their school refusal behaviors, it is possible that when adolescents are not ready to regulate emotional difficulties on their own, they rely on their parents to manage distress related to school refusal behaviors. Indeed, the neural regions in the prefrontal cortex that underlie the regulation of emotion may not reach full maturity until late adolescence (Spear, 2000). Furthermore, the normal course of developing emotion regulation skills requires an understanding of how emotions work and what emotion regulation strategies are most effective in certain situations (Hannesdottir & Ollendick, 2017). If readiness is lacking, adolescents may remain overly dependent on their parents for assistance in regulating emotions instead of doing it on their own. This way, the ineffective emotion regulation strategies provided by their parents may translate into low perseverance with attempts to enforce school attendance.

Finally, inconsistent with Ingul et al. (2019), the relationship between adolescent anxiety and depression and their school refusal behaviors was not significant when parental emotion dysregulation was considered in the model, although it was approaching significance (*p* = .052). This could be due to the relatively small sample size of the current study (*N* = 106) to provide good estimates of all of the factors and terms being included in the model. Future research should include a bigger sample to consolidate the conclusions.

### Limitations and Future Directions

The current study is not without limitations. First, the cross-sectional design means it is not possible to determine the direction of the relationship between parental and adolescent factors and school refusal behaviors—reciprocal relationships are likely involved. A future longitudinal study could address this limitation. Furthermore, based on the recruitment procedure, the current study might not have included all students with severe school refusal behaviors given this population of students is often more disengaged from school attendance and activities. The global understanding of adolescents with school refusals is also limited by the lack of information on ethnicity and the impacts of disabilities in the current study (Allen et al., 2018; Nordin et al., 2023). For example, non-English speaking students and autistic students are often at a higher risk of engaging in school refusal behaviors, which is likely to exacerbate stress-related factors among parents. Similarly, the inclusion of socio-economic factors in the study (such as family income or parental education status) could have provided an additional opportunity to enrich control for the influence of family socio-economic status or to explore potential moderating differences (Sosu et al., 2021). Finally, it is difficult to ascertain the extent to which our findings may be influenced by social desirability bias in the measurement of parental rearing styles. Thus, the use of a range of parenting instruments is needed in future research. Positively, this study included information from both parents and children, with a unique combination of factors tested and a robust measure of school refusal behaviors that incorporated both administrative reports from the school and qualitative checking from the adolescents.

### Theoretical and Clinical Implications

This study is one of the very few that have comprehensively investigated the role of parental and adolescent factors in school refusal behaviors. This is a dire need given the current limited knowledge about evidence-based and effective interventions for school refusal behaviors in adolescents (Elliott & Place, 2019). Our findings establish a significant role of parental emotion dysregulation in school refusal behaviors, which is important for assisting an integrative understanding of school refusal behaviors in adolescents, and in turn, contributes to improving their school and daily functioning. Future research should examine whether the current findings also apply to children, which could help establish more population and age-tailored theoretical frameworks and interventions.

The current study also provides insight into the development of future interventions for adolescents with school refusal behaviors. Our results suggest that management of school refusal behaviors in adolescents should consider their age differences and develop age-tailored approaches. In addition, interventions for school refusal behaviors should also integrate components that target parental emotion dysregulation. Specifically, management protocols and support systems should involve parents and include strategies that assist parents in developing adaptive emotion regulation strategies to help increase adolescents’ attendance in school. Furthermore, considering the lifelong negative outcomes associated with frequent school absences (e.g., lower income, frequent work absences, and poor health), equipping parents with effective emotion regulation strategies will enable them to become integral members of a supportive network. This involvement will facilitate the early identification of children in need of assistance and aid in the prevention of relapses into school refusal behaviors (Ingul et al., 2019; Kearney, 2022).

## Conclusions

Frequent school absences have both immediate and long-term negative effects on youths’ academic, social, and health functioning (Allen et al., 2018; Kearney, 2022). Thus, a comprehensive understanding of this condition, employing an integrative perspective that considers interrelated factors, is vital for grasping its complexity. The present study examined both adolescent and parental factors that are associated with adolescents’ school refusal behaviors, using data from both parents and their child. Our results contribute to the literature by demonstrating the important role of parental emotion dysregulation and adolescent age in potentially impacting school refusal behaviors. Future management for adolescents’ school refusal behaviors should consider adolescents’ age and assist parents in gaining adaptive emotion regulation strategies to help adolescents manage their school refusal behaviors.

## Supplemental Material

sj-docx-1-bmo-10.1177_01454455241276414 – Supplemental material for School Refusal Behaviors: The Roles of Adolescent and Parental Factors Supplemental material, sj-docx-1-bmo-10.1177_01454455241276414 for School Refusal Behaviors: The Roles of Adolescent and Parental Factors by Junwen Chen, Celina Feleppa, Tingyue Sun, Satoko Sasagawa, Michael Smithson and Liana Leach in Behavior Modification

## Notes

1. We use “youth” when referring collectively to children and adolescents aged 5 to 17 years, and when there is no clear specification about children and adolescents in the articles we cited. When referring to a specific developmental level, we use “child” or “adolescent.”
