# Supplementary material for: School Refusal Behaviors: The Roles of Adolescent and Parental Factors
Source: Behav Modif. 2024 Aug 26;48(5-6):561–80. doi: 10.1177/01454455241276414 (PMC11497732; doi:10.1177/01454455241276414)
Supplement: sj-docx-1-bmo-10.1177_01454455241276414 – Supplemental material for School Refusal Behaviors: The Roles of Adolescent and Parental Factors [file sj-docx-1-bmo-10.1177_01454455241276414.docx]

**Supplementary Material 1.**

*The criteria for school non-attendance by the Department for Education (Former Department for Education and Child Development) in South Australia (2017-2020):*

| **Habitual non-attendance** | Where a student has 5 or more absences for any reason in a term (average of 1 day per two weeks) |
| --- | --- |
| **Chronic non-attendance** | Where a student is absent for 10 days or more in a term for any reason (average of 1 day per week) |

Based on the criteria, the criterion for identifying “school refusal” in the current study is as below, which counts the number of school days on which a school refusal behaviour occurs:

| **School refusal** | Where a student is absent for 10 days or more in a term for any reason (average of 1 day per week). |
| --- | --- |

**Supplementary Material 2.**

*Examples of Criteria for School Refusal (Behaviours)*.

| **Reference** | **Purpose of Study** | **Criteria for School Refusal Behaviour** | **Average No. of Days Absent** |
| --- | --- | --- | --- |
| Elliott, J. G., & Place, M. (2019). Practitioner review: school refusal: developments in conceptualisation and treatment since 2000. *Journal of Child Psychology and Psychiatry*, *60*(1), 4-15. | Review paper - to identify progress subsequently made this century on the conceptualisation, assessment, and treatment of school refusal | There are multiple variations in the literature for what is considered to be defining features of school refusal behaviour. Definitions in the literature have included any of the following: 1) Less than an 80% attendance record over the previous 2 weeks 2) The presence of an anxiety disorder as identified in DSM-IV (excluding OCD and PTSD) 3) Parents could account for the whereabouts of the child on the days marked by school absence 4) No concurrent DSM-IV conduct disorder  5) Clear commitment on the part of parents to help the child to achieve full school attendance except when for legitimate reasons. | Less than 80% attendance over the past 2 weeks (average: 3 days absent per two weeks) |
| Heyne, D. (2019). Developments in classification, identification, and intervention for school refusal and other attendance problems: introduction to the special series. *Cognitive and Behavioral Practice*, *26*(1), 1-7. | Review paper – Review of 7 research papers to address two key questions: (1) How can school attendance problems best be classified and identified; and (2) Which innovative approaches can be applied when a school attendance problem is severe, chronic, and/or complex? | There is little indication of an emerging consensus on the conceptualisation and classification of school refusal behaviour. Definitions of school refusal behaviour vary greatly between countries and school communities. Research has reported on the following: 1) Kearney’s (2008) absence criteria to identify youth with problematic absenteeism (i.e., at least 25% absence for 2 or more weeks or at least 15% absence across 15 school weeks)  2) Reporting on the prevalence of absenteeism based upon a minimum absence criteria (e.g., at least 1 day) rather than identifying the percentage of youth displaying problematic absenteeism  3) Considering the function of school refusal behaviour more so than the classification by using the School Refusal Assessment Scale (Kearney, 2002). | At least 25% absence for 2 or more weeks (average: 3 days absent per two weeks)  At least 15% absence across 15 school weeks (average: 11-12 days absent in 15 weeks) |
| Ingul, J. M., Havik, T., & Heyne, D. (2019). Emerging school refusal: a school-based framework for identifying early signs and risk factors. *Cognitive and Behavioral Practice*, *26*(1), 46-62. | Review paper - this paper provides discussed the likely signs and risks for emerging school refusal. A school-based framework is provided to support school personnel and parents in working together to identify these signs and risks. | School refusal behaviour can take many forms, including: 1) Late arrival from school 2) Early dismissal from school and/or 3) Total absence from school  On average, youth were considered to have emerging school refusal behaviours when they were absent for 4.2 half days or 2 days of absence across 3 months | At least 4.2 half days or 2 full days of absence across 3 months |
| Balakrishnan, R. D., & Andi, H. K. (2019). Factors associated with school refusal behaviour in primary school students. *Muallim Journal of Social Sciences and Humanities*, 1-13. | This study aimed to identify the factors associated with school refusal behaviour in primary school students. | All students who have a documented history of 15% or more of unexcused absences during the 2016 school year were asked to participate in this study. | At least 15% of unexcused absences in the year (approximately 30 days absent in the year) |
| Carroll, H. C. M. (2020). The relative effect of pupil absenteeism on literacy and numeracy in the primary school. *Educational Studies*. <https://doi.org/10.1080/03055698.2020.1793302> | This paper investigated the possible causes and effects of pupil absenteeism in the primary schools. | All the Year 6 pupils with attendance rates of 80% or less in both Years 2 and 6 | Less than 80% attendance over the past 2 weeks (average: 3 days absent per two weeks) |
| Havik, T., Bru, E., & Ertesvag, S. K. (2015). School factors associated with school refusal- and truancy-related reasons for school non-attendance. *Social Psychology of Education*, *18*(2), 221-240. <https://doi.org/10.1007/s11218-015-9293-y> | The primary aim of this study was to investigate how students’ perceptions of relationships with peers at school and teachers’ classroom management are associated with school refusal-related reasons and truancy-related reasons for school non- attendance. | Students with more than 1 day of absence in the past 3 months were invited to participate. | More than 1 day of absence in past 3 months |
| Jones, A. M., & Suveg, C. (2015). Flying Under the Radar: School Reluctance in Anxious Youth. *School Mental Health*, *7*(3), 212-223. <https://doi.org/10.1007/s12310-015-9148-x> | This study examined associations among school reluctance, somatic complaints, impairment, and social and emotional functioning in a sample of youth diagnosed with one or more anxiety disorders. | School refusal was defined as missing greater than 20% of days of school in a given school year | Greater than 20% absence from school in a year (40 days per year absence) |
| Kearney, C. A. (2008a). An interdisciplinary model of school absenteeism in youth to inform professional practice and public policy. *Educational Psychology Review*, *20*(3), 257-282. <https://doi.org/10.1007/s10648-008-9078-3> | This article reviewed literature from psychology, social/criminal justice, and education to outline important factors that influence school absenteeism. | Problematic absenteeism could refer to school-aged youths who: (1) have missed at least 25% of total school time for at least 2 weeks (2) experience severe difficulty attending classes for at least 2 weeks with significant interference in a child’s or family’s daily routine, and/or  (3) are absent for at least 10 days of school during any 15-week period while school is in session (i.e., a minimum of 15% days absent from school). | At least 25% absence for 2 or more weeks (average: 3 days absent per two weeks)  A minimum of 15% days absent from school in a 15-week period (10 days absence from school in 15 weeks) |
| Maric, M., Heyne, D. A., MacKinnon, D. P., Van Widenfelt, B. M., & Westenberg, P. M. (2013). Cognitive mediation of cognitive-behavioural therapy outcomes for anxiety-based school refusal. Behavioural and cognitive psychotherapy, 41(5), 549-564. | This study aimed to investigate the role of self-efficacy in mediating Cognitive Behaviour Therapy outcomes for anxiety-based school refusal. | To qualify as school refuser: 1. Attend school less than 80% of the time 2. DSM-IV anxiety disorder 3. Parents could account for the adolescent’s whereabouts on days absent 4. No current DSM-IV conduct disorder 5. Current enrolment in school | Less than 80% attendance (minimum 1 day absence per week, 2 days per two weeks) |
| Munkhaugen, E. K., Gjevik, E., Pripp, A. H., Sponheim, E., & Diseth, T. H. (2017). School refusal behaviour: Are children and adolescents with autism spectrum disorder at a higher risk? *Research in Autism Spectrum Disorders*, *41*, 31-38. | This study assessed the frequency of school refusal behaviour in students with ASD aged 9–16 years without intellectual disability (IQ > 70) compared to typically developing (TD) students. | A questionnaire based on Kearney’s description of school refusal was developed for this study. School refusal behaviour was defined as 1–20 days of refusal to attend school/classes expressed verbally or physically, or as partial or complete unauthorized absenteeism. | Between 1-20 days of refusal to attend school in the past month |

**Supplementary Material 3.**

*Correlations between Adolescents and Parental Factors*

|  | *1* | *2* | *3* | *4* | *5* | *6* | *7* | *8* | *9* | *10* | *11* | *12* |
| --- | --- | --- | --- | --- | --- | --- | --- | --- | --- | --- | --- | --- |
| **Adolescents** |  |  |  |  |  |  |  |  |  |  |  |  |
| 1. Anxiety | 1.00 | .66** | .26** | -.16 | .29** | .03 | .01 | .04 | -.03 | .04 | .11 | -.20* |
| 1. Depression | .66** | 1.00 | .25* | -.16 | .27** | .06 | .14 | .13 | -.03 | .10 | -.06 | -.10 |
| 1. Ex_Sup | .26** | .25* | 1.00 | .08 | .48** | -.13 | .04 | .25* | .01 | .15 | -.12 | .12 |
| 1. Cog_ReAp | -.16 | -.16 | .08 | 1.00 | -.84** | -.21* | -.13 | -.05 | .06 | -.08 | .04 | .03 |
| 1. Total_EdR | .29** | .27** | .48** | -.84** | 1.00 | .12 | .14 | .18 | -.05 | .15 | -.10 | .09 |
| **Parent** |  |  |  |  |  |  |  |  |  |  |  |  |
| 1. Anxiety | .03 | .06 | -.13 | -.21* | .12 | 1.00 | .68** | .07 | -.33** | .28** | -.02 | .02 |
| 1. Depression | .01 | .14 | .04 | -.13 | .14 | .68** | 1.00 | .16 | -.46** | .43** | -.09 | -.04 |
| 1. Exp_Sup | .04 | .13 | .25* | -.05 | .18 | .07 | .16 | 1.00 | -.11 | .71** | -.22* | .28** |
| 1. Cog_ReAp | -.03 | -.03 | .01 | .06 | -.05 | -.33** | -.46** | -.11 | 1.00 | -.78** | .24* | .08 |
| 1. Total_EdR | .04 | .10 | .15 | -.08 | .15 | .27** | .43** | -.71** | -.78** | 1.00 | -.31** | .11 |
| 1. RS_Nurt | .11 | -.06 | -.12 | .04 | -.10 | .82 | .32 | -.22* | .24* | -.31** | 1.00 | -.25* |
| 1. RS_Rest | -.20* | -.10 | .12 | -.03 | .09 | .02 | -.04 | .28** | .08 | .12 | -.25* | 1.00 |

*Note.* Exp_Sup = Expressive Suppression. Cog_ReAp = Cognitive Reappraisal. Total_EdR = Total Emotion Dysregulation. RS_Nurt/Rest = Rearing Style Nurturance/Restrictiveness. * *p* < .05; ** *p* < .01; ****p* < .001 (two tailed tests)

**Supplementary Material 4.**

*Adolescent Emotion Dysregulation, Anxiety and Depression*

We tested whether adolescent emotion dysregulation, adolescent anxiety and depression were associated with high odds of adolescents’ school refusal behaviours (see Table below). In Step 1, adolescent age was significantly associated with school refusal behaviours. The odds of adolescents having school refusal behaviours were 1.49 times higher (95% CI = [1.14, 1.94]) when there was an increase of 1 standard deviation in adolescent age. In Step 2, adolescent emotion dysregulation was not significantly associated with school refusal behaviours. However, adolescent anxiety and depression composite score entered in Step 3 was significantly associated with school refusal behaviours. The odds of adolescents with school refusal behaviours were 1.67 times higher (95% CI = [1.04, 2.67]) when there was an increase of one standard deviation in adolescent anxiety and depression. The likelihood-ratio test comparing the models in Steps 2 and 3 showed a significantly improved fit in the Step 3 model (*c* ^2^ (1) = 4.60, *p* < .05). Both adolescent age and adolescent anxiety and depression were significantly associated with adolescents’ school refusal behaviours. To further examine whether there were joint effects of adolescent age, and adolescent anxiety and depression on adolescents’ school refusal behaviours, the correlation between adolescent age, and adolescent anxiety and depression composite scores was examined. The result was not significant (*r* = .18, *p* = .07). Further, the product term of adolescent age and adolescent anxiety and depression was created. Adding it to the model did not significantly improve the model fit (*ꭓ*^2^ (1) = 0.80, *p* = .37).

*The Relationships between Adolescent Emotion Dysregulation, Anxiety and Depression, and School Refusal Behaviours*

| Model | Variables | *B* | SE | Wald’s *c*2 | *p* | Exp(*B*) | CI | |
| --- | --- | --- | --- | --- | --- | --- | --- | --- |
|  |  |  |  |  |  |  | Lower | Upper |
| Step 1 | Adolescent Age | 0.40 | .13 | 8.72 | .003** | 1.49 | 1.14 | 1.94 |
| Step 2 | Adolescent Age | 0.43 | .14 | 9.52 | .002** | 1.54 | 1.17 | 2.02 |
|  | Adolescent Emotion Dysregulation | 0.45 | .24 | 3.54 | .06 | 1.57 | 0.98 | 2.52 |
| Step 3 | Adolescent Age | 0.39 | .14 | 7.68 | .006** | 1.48 | 1.12 | 1.96 |
|  | Adolescent Emotion Dysregulation | 0.32 | .26 | 1.52 | .22 | 1.38 | 0.83 | 2.28 |
|  | Adolescent depression+anxiety | 0.51 | .24 | 4.51 | .03* | 1.67 | 1.04 | 2.67 |

*Note. B* = unstandardised regression coefficients; SE = standard error; CI = confidence interval.

* *p* < .05; ** *p* < .01; ****p* < .001 (two tailed tests)
